# Combined pelvic ring and acetabular fractures – strategies and sequence of surgery. State of the art

**DOI:** 10.1007/s00402-024-05555-4

**Published:** 2024-09-23

**Authors:** Paul Puchwein, Gunnar Sandersjöö, Jan Lindahl, Nicolas Eibinger

**Affiliations:** 1 Department of Orthopedics and Trauma Surgery, Medical University of Graz, Graz, Austria; 2 Department for Trauma Acute Surgery Orthopedics, Karolinska University Hospital, Stockholm, Sweden; 3 Department of Orthopaedics and Traumatology, Helsinki University Hospital, Helsinki, Finland

**Keywords:** Pelvic ring fracture, Acetabulum fracture, Combined pelvic ring injury

## Abstract

Combined injuries of the pelvic ring and the acetabulum are uncommon. Acute treatment should follow common protocols (ATLS e.g.) for pelvic ring injuries, although mechanical stabilization using pelvic binders or external fixators might be insufficient or even worsen the reduction in some combined fracture patterns. In case of mechanically connected acetabular and pelvic ring injury (MCAPI), surgical treatment might be demanding in lack of clear recommendations concerning the reduction and fixation sequence. A “pelvic ring first” sequence may be the best choice for most MCAPIs, starting with sacrum or SI-joint and symphysis pubis. An “acetabulum first” sequence should be considered in relatively stable posterior ring injuries and acetabulum fractures in younger patients, where a perfect anatomical reduction is feasible. Definitive surgical treatment should be performed as soon as possible depending on concomitant injuries, ideally within 3–7 days. Mechanical understanding of the combined fracture pattern and accurate planning are mandatory for surgical repair.

## Introduction

Uni- or bilateral combined injuries of the pelvic ring and the acetabulum have a relatively low incidence with 9% of all acetabular fractures and 5-16% of all pelvic ring fractures Severe pelvic trauma with combined acetabulum fractures rarely presents as single injuries, concomitant injuries of the CNS, thorax and/or abdomen are frequent [1–8].

Due to a lack of quantity and quality of literature there is still discussion about what is the main goal of reduction and fixation in combined injuries. There is also uncertainness concerning the types of fractures that should be included in the term “combined injuries”. The lack of discrimination between high pubic ramus fractures and low anterior column fractures, e.g., makes it difficult to compare cohorts if latter are also included in “combined injuries” [1]. Another vagueness in the definition of combined injuries is a both column fractures with a “floating” acetabulum, which should also not be included in the narrow definition of such injuries [9].

Some authors give more importance to the reduction of the acetabular fracture, while others are focusing on the acetabular component [1, 5]. There are several other factors affecting the overall outcome of such complex injuries like age of the patient, comminution elements in acetabular fracture (posterior wall e.g.), dislocation of the femoral head anatomic reducibility especially of the acetabular fracture and preexistent osteoarthritis.

The predominant cause of injury in adult patients is a motor vehicle accident, followed by pedestrians, hit by motor vehicles [1, 10]. Similar trauma mechanisms were observed in pediatric patients, whereat most children were injured as car occupants or as pedestrians [11]. Injuries of the triradiate cartilage were seen in 30% of their patients with isolated and combined injuries, but with 36% the amount of triradiate cartilage injuries was higher in the combined cohort. According to the AO classification most pelvic ring injuries in pediatric patients were classified as B2 and B3. Operative management was associated especially with injuries involving posterior wall [11].

The term “mechanically connected acetabular and pelvic ring injuries” (MCAPI) was introduced by the authors to discriminate clearly between injuries in which reduction and fixation of one fracture affects the other, and simply coexisting and non-interacting injuries of acetabulum and pelvic ring (e.g.: posterior wall and B2 injury, acetabulum and pelvic ring on different sides) – Non-MCAPI.

The purpose of this review is to summarize recent literature and to give clear recommendations how to treat such complex injuries.

## Imaging and classification

While conventional radiographs may be helpful in obtaining a first overview about the injury pattern, a multislice CT scan (with 3D reconstruction if available) is the gold standard and mandatory in such (poly-)trauma cases to classify the fracture and detect relevant concomitant injuries (Tone19). Difficulties in evaluation of scant literature are the different classifications used for pelvic ring and acetabulum fractures. The authors decided to use the Judet/Letournel classification for acetabular fractures and the AO/OTA classification for the pelvic ring, as latter combines Tile and Young Burgess classifications [12, 13]. The most common acetabular fracture pattern in combined injuries in adults are transverse (21–61%), both columns (14–25%), transverse with posterior wall (7–17%), anterior column posterior hemi-transverse (11–13%), T-shape (8–23%) and anterior column fractures (4–18%) [2, 7, 10, 14].

In pediatric patients the prevalent acetabular fracture for operative treatment is also posterior wall, the most frequent conservatively treated pattern is anterior column or anterior wall, depending on authors’ decision if they have included high roots ramus fractures in their definition of combined injuries [11].

MCAPI can include following acetabular patterns: anterior column, transverse-types, T-shape-types and both columns. Isolated posterior wall, posterior column or combination of both, as well as anterior wall fractures may be treated as independently to the ring fracture in our opinion (Non-MCAPI). Concerning the pelvic ring pattern, all AO/OTA B2,3 and C1-3 injuries should be included, as the usually have noticeable dislocations and need any kind of surgical fixation (see Table 1). MCAPI and Non-MCAPI can be accompanied by femoral head dislocation (posteriorly in PW fractures, centrally in any kind of transverse fractures), which has to be addressed separately.

In children, an MRI after reduction of a dislocated hip is recommended to identify non osseus indications for surgery [11].

The predominant fracture pattern of the pelvic ring component is an AO/OTA B-injury in 63–69%, followed by C-injuries in 31–37% [2, 15]. Bilateral MCAPIs are very rare (0.06%) [16].

### Acute treatment

**Fig. 1:**
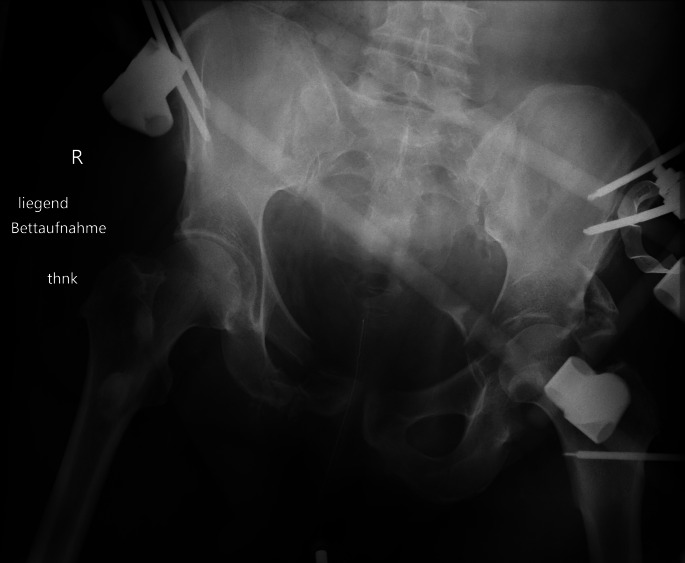
MCAPI, pelvic ring OTA/AO C2.2, acetabulum TrPW (B1.2). Cyclist hit by car, complete avulsion of lumbosacral plexus. External fixator used for acute fixation of pelvic ring, joint bridging fixator pin to keep reduction of displaced femoral head and to fix a femoral shaft fracture on the left side (floating acetabulum)

Initial assessment and acute care in emergency room follows common algorithms like ATLS^®^ [1]. In hemodynamically unstable patients a Damage Control approach will be the choice. The main target in this phase is to stop the bleeding by closed reduction and in some cases mechanical stabilization of the pelvic ring. For provisional stabilization of type B open book injuries and type C pelvic ring injuries pelvic binders (PCCD) are most often used, external fixators very rarely in the early face. For non-responders, preperitoneal pelvic packing (PPP) and/or angioembolization and/or REBOA are the current options [1, 5]. Nevertheless, routine technics for reduction of pelvic ring injuries or displaced femoral heads in dislocated acetabular fractures might be ineffective or fail in case of MCAPIs [5, 6]. A combination of different techniques might be helpful in some cases (see Fig. 1).

The use of mechanical stabilization devices in pelvic fractures is varies in different countries. Whereas in central Europe pelvic binders are used routinely in pelvic fractures, Sweden e.g., is very restrained in the use of such strategies. Actually, there is no high evidence showing an advantage in mortality in pelvic injuries due to the use of any stabilization devices [17]. Recent studies show no advantage of PCCDs in terms of mortality or need for blood transfusions [18], no difference in short- and long-term mortality between external fixator and PCCDs [19] and a high rate (up to 39%) of incorrectly applied PCCDs [20], that could probably worsen the outcome. The general use of mechanical stabilization devices has to be scrutinized and should be reserved for selected cases.

However, concomitant injuries of the pelvic floor including urinary tract injuries, the rectum, soft tissue injuries (Morell-Lavallée, e.g.) or neurovascular injuries (tear of the lumbosacral plexus and or iliac veins/arteries, e.g.) must be detected early and included in the damage control strategy. Salvage procedures like hemipelvectomies might also be considered in patients in extremis to safe their life (see Figs. 4 and 5). Combined injuries should be treated in the highest available trauma center. Concerning mortality, a RR of 0.3 at 90 days and after one year could be demonstrated, when patients were treated in Level-I-trauma centers [21].

### Definitive treatment

#### Conservative treatment

A conservative therapeutic approach even for combined injuries was common till the 1970ies, but even in the 1990ies more than 50% of B- and C-type fractures were treated conservatively [8].

Despite the discussion which fractures should be included in combined injuries, conservative treatment might be the right choice for undisplaced acetabular fractures and relatively stable pelvic ring fractures (AO/OTA A1-3, B1). Conservative treatment of the pelvic ring injury might be considered in a T-type fracture with widening of the SI-joint, when there is finally congruency in the SI-joint after ORIF of the acetabulum.

In our opinion there are several reasons for conservative treatment: Fractures that allow early mobilization with conservative treatment (Non-MCAPIs like undisplaced PW fracture with Zone 1 sacrum fracture, e.g.), a serious condition of the patient that does not allow surgery (traumatic brain injury with high ICPs, e.g.) or acceptable dislocation of unstable fractures without any advantage of surgery, because early weight bearing is not advisable due to other conditions (relevant injuries of the lower limbs not allowing weightbearing).

Patients with conservatively treated acetabular fractures may show better functional outcomes (62% excellent/good) compared to radiological findings (23% excellent/good) [22]. The main problem of conservative treatment of displaced fractures is the need for an 8-week traction therapy with Steinmann-pins or Schanz-pins in the greater trochanter with a relevant risk of complications due to the long immobilization period (35–61% of immobilized pelvic patients suffer deep venous thrombosis), so that this therapeutic option should be reserved for inoperable patients [22, 23].

#### Surgical treatment and timing

**Fig. 2:**
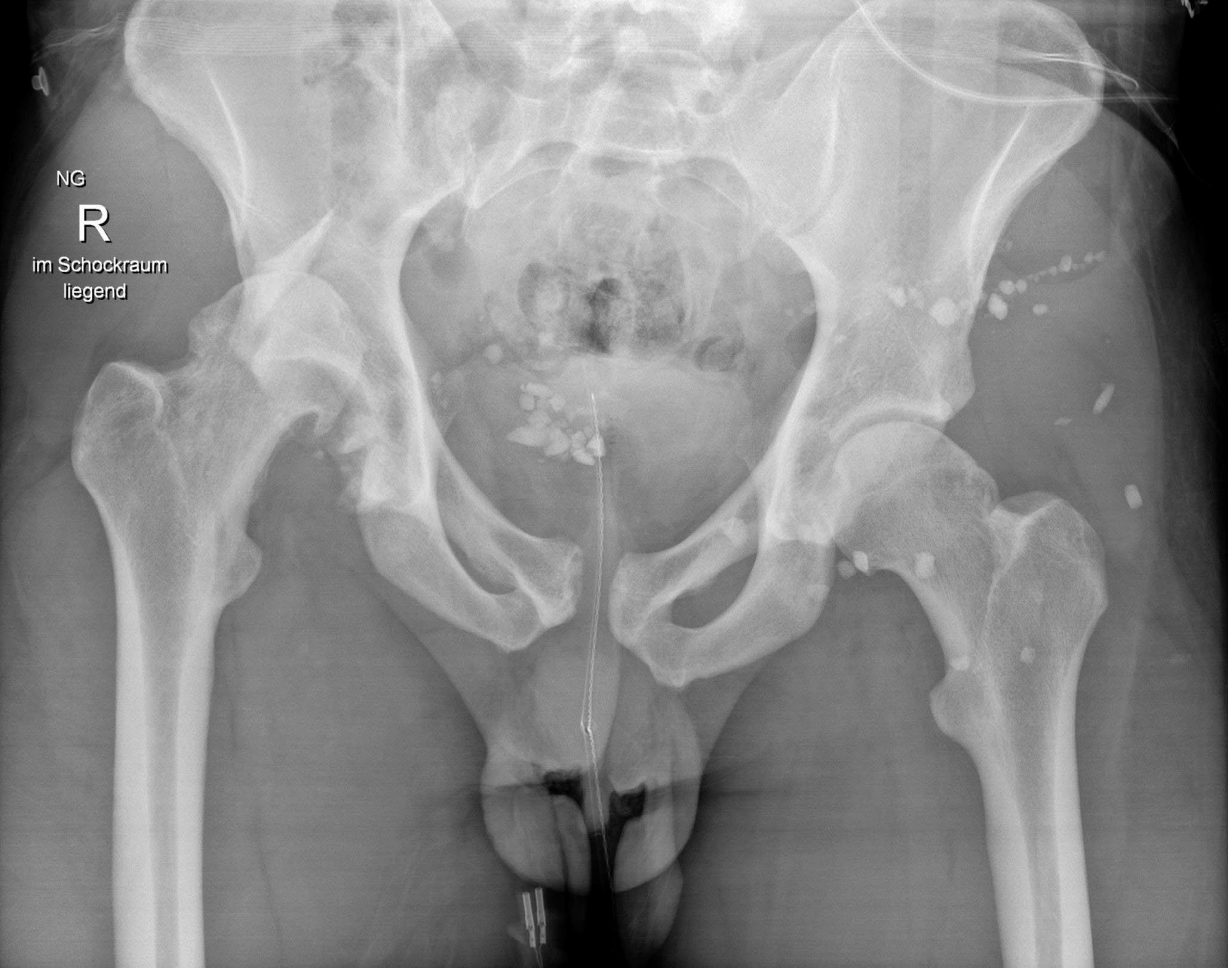
Non-MCAPI, pelvic ring OTA/AO C1.2 L, acetabulum PW (A1.2). Fall from 20 m. Concomitant injuries: lung contusions bilateral. Hemodynamically stable patient

**Fig. 3:**
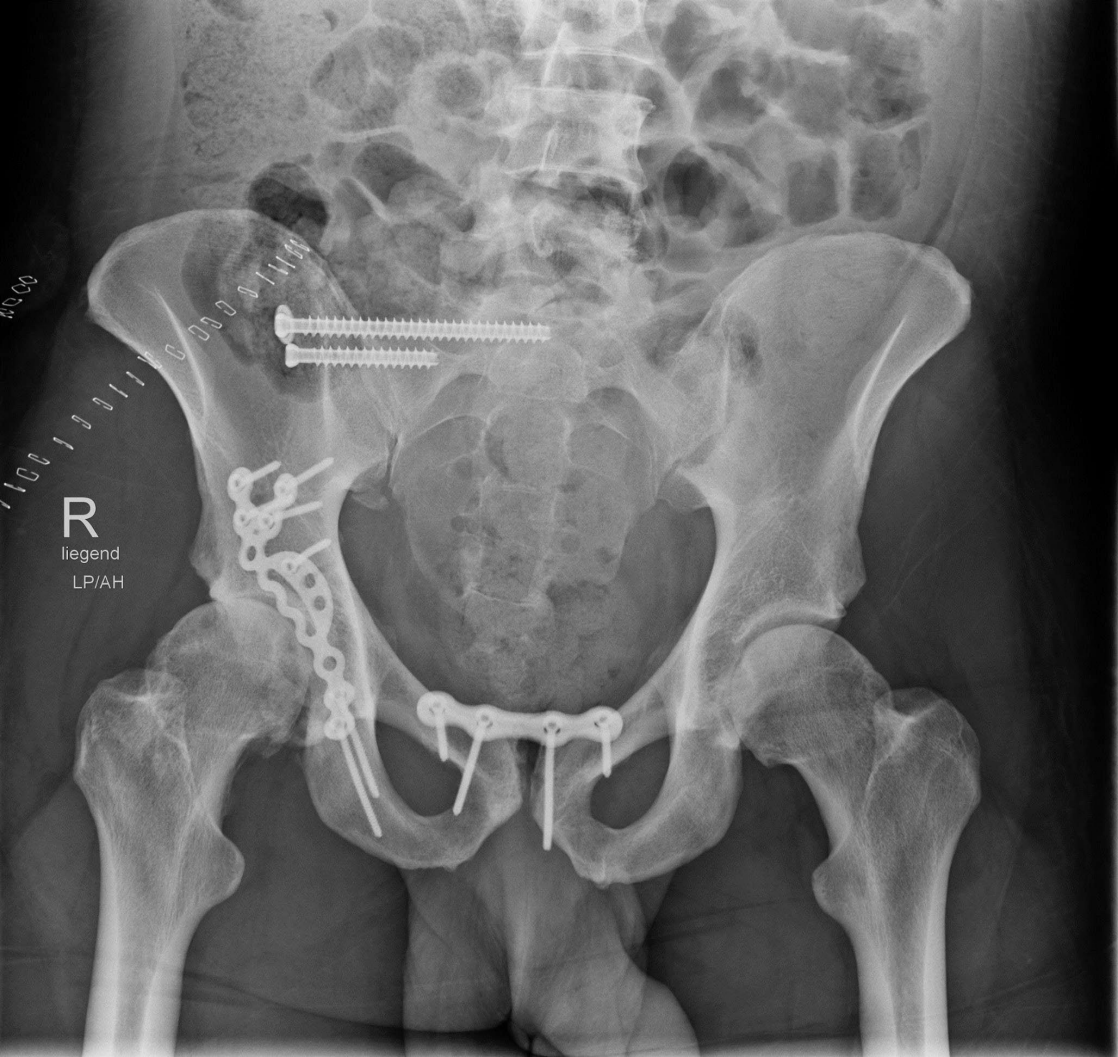
Early total care, first repair of PW fracture and SI screws in prone position, then fixation of anterior pelvic ring. Patient was extubated after surgery and left ICU the next day, no lung or other complications

The terms “early” and “late” are not clearly defined in literature, what makes it difficult to compare different studies [23]. Associated injuries are the determinant concerning the timing of definitive pelvic and acetabular repair. But against former opinions for a delayed timing, recent literature shows advantages of an early repair [5]. It could be demonstrated that patients who were operated within 14 days had fewer rates of acute respiratory distress syndromes or other pulmonary complications, less organ failures, shorter hospital stays, fewer transfusions and a reduced mortality [5]. Other authors define “early” from 8 h to 7 days and recommend surgical fixation after 72 h to avoid excessive blood loss to early after trauma [24]. They found a decrease from 125 to 38 h in average for definitive repair of pelvic ring fractures in the last decade, and from 160 to 33 h for polytrauma subgroup without any increase of complications [24]. Though 3 to 7 days seems to be a safe time window for pelvic ring and acetabular repair, there are selected patients were early total care could be considered [2] (see Fig. 2 & 3). Late repair results in worse reduction and results [23].

Planning the right sequence in MCAPIs, it should be considered, that a displacement of the posterior ring of < 1 cm can be accepted, whereas acetabular fractures should not exceed a displacement of > 2 mm [5, 25]. By using extensible approaches like the intrapelvic approach with lateral window, the ilioinguinal approach or the pararectus approach, reduction and fixation can be achieved for both injuries. Due to the need of a prone or lateral decubitus, a posterior Kocher-Langenbeck approach has more limits in combined injuries, tough a combination with an anterior approach by using a “floating position” could be an option.

Another technique that could be useful especially in combined injuries is an intraoperative or temporary internal fixator (INFIX) [4].

In fact, every combination of both injuries requires a careful planning and decision on approaches and positioning of the patient. In the following paragraph the authors offer some recommendation for both types of surgical sequences depending on fracture types and patient factors. In fact, revision surgery of the hip joint in case of secondary arthrosis due to inaccurate reduction by THA is easier than revision of pelvic ring non unions or malunions. Our aim was to identify factors that may prioritize acetabulum or the pelvic ring.

#### “Acetabulum First” – sequence (AFS)

The fact, that the acetabulum needs a higher accuracy of reduction should not mislead the surgeon to start there in any case, which is recommended by some authors [1].

There are some fracture patterns, in which the AFS may be the easier one. Transverse fractures with a B2.3 pelvic ring injury (APC2) should be addressed in this way (see Fig.6). Usually the SI-joint will be nearly congruent after acetabulum repair. Percutaneous screw fixation or conservative treatment for the posterior pelvic ring can be considered then [5].

Another indication for AFS are cases in which percutaneous screw fixation for the acetabulum is indicated, followed by fixation of the pelvic ring [2].

In younger patients with displaced acetabular fractures anatomic reducibility is a requirement for AFS.

#### “Pelvic Ring First” – sequence (PFS)

In most cases with relevant dislocation of the pelvic ring fracture, a PFS should be preferred. An incomplete reduction of the posterior ring might result in an unsatisfactory reduction of the acetabular fracture, especially in all types of transverse fractures [1, 15].

Displaced type B and C pelvic ring fractures have the highest level of posttraumatic chronic pain [15]. In opposite to the pelvic ring, failed acetabular fixations can be easily addressed with total hip arthroplasty (THA). Contrary to isolated pelvic ring fractures the surgeon is not able to reduce the posterior ring sufficiently by anterior procedures in the presence of a MCAPI due to the interrupting fracture of the acetabulum. Therefore a sequence starting posteriorly should be preferred [15].

PFS is also advisable in bilateral crescent fractures (AO/OTA B2.2), the type of fixation of the crescent fragment depends on the size and fracture type [26]. Transverse and transverse variant fractures with ipsilateral SI-joint injuries can also be managed by PFS with excellent results [27]. In a case of a bilateral transverse fracture and bilateral open SI joints, S1 corridor screws were placed on both sides in combination with an external fixator, resulting in a complete reduction of the acetabular fractures on both sides followed by conservative management [16]. Another helpful tool could be a temporary INFIX, especially in comminuted anterior ring or open fractures [4].

Fixation of anterior pelvic ring before fixing the acetabulum could make the reduction more difficult. This is contrary to isolated pelvic ring fracture, were anterior reduction could be the first step. Posterior ring should be addressed first, anterior ring fixation should be the latest in the fixation sequence [6]. Total disruption and displacement of the symphysis pubis is an exception to this. In a series of BC acetabular fractures PFS was superior to AFS in terms of blood loss and time for surgery [9].

### Aftercare

Early mobilization in a wheelchair and/or at least partial weight bearing should be the goal in combined injuries to prevent complications, depending on if the injury was uni- or bilateral. Thrombosis prophylaxis with LMWH or NOACs is recommended for 6–8 weeks [28]. Partial weight bearing using crutches should be started not later than 8 weeks after surgery, full weight bearing not later than 12 weeks postoperatively [2].

### Outcome and complications

Poor outcomes may occur in more than 20% of all patients due to a deficient reduction of acetabular fracture, pelvic ring fracture or both, sciatic nerve injuries (iatrogenic or traumatic), surgical site infections and heterotopic ossification [1, 4]. An overall risk for complications up to 44% is reported for combined injuries [14]. C-injuries (reduced anatomically in 57%) of the pelvic ring have a higher risk of a malreduction than B-injuries (reduced anatomically in 84%) [1, 8]. Anatomic reduction of the acetabular fracture has been achieved in only 33% [2].

Overall mortality reaches from 8 to 52% for combined injuries, isolated pelvic ring fractures have a higher mortality rate than isolated acetabulum fractures [1, 5, 10].

Hip dislocation at primary survey is associated with significantly increased complications at final follow-up. Concomitant fractures of the lower extremities prolong time to ORIF, whereas a delayed ORIF is associated with a higher complication rate [14]. Neurological deficits are present in 36% of patients with combined injuries, another 4–6% are caused iatrogenically, up to 16% can persist permanently [2, 14].

Surgical site infections are more likely in patients with open acetabular approaches than in percutaneous techniques [14]. Moreover, infection risk is higher in patients treated with external fixators and patients which received angioembolization and a higher BMI, and lower in patients with selective angioembolization, fewer intraoperative transfusions and shorter surgery time [3, 5]. Quality of reduction might be worse in fractures operated > 8 days and reduction is definitively worse when ORIF is performed after 3 weeks [5].

Acetabular malalignement is higher (2.6 mm) in combined injuries than in isolated acetabulum fracutre (1.1 mm) [29]. Mean malalignement of the posterior ring is 3.5 mm [2].

In pediatric patients hip dysplasia, leg length discrepancy and (rarely) premature closure of the triradiate cartilage are the main complications in combined injuries. Infections, nonunions and fixation failures are no issues in the pediatric cohort [11]. Deep venous thrombosis occurs in up to 13% of patients [2]. Bony healing can be expected after 15 weeks [2]. Concerning complications of surgical approaches in all pelvic ring and acetabular fractures, the highest complication rate is reported for the use of external fixators (31%, thereof 22% infections), the intrapelvic approach (17%), percutaneous approaches (11%) and the ilioinguinal approach (7%) [30]. The risk of neurological complications is highest in the Kocher-Langenbeck approach (5.5%) compared to the intrapelvic approach (1.1%) [30].

Overall, neurological complications like sciatic nerve palsy or injuries of the lumbosacral plexus, infections and heterotopic ossifications are determinants for worse outcomes [4].

In case of comminution, elderly patients or unreducible acetabular fractures the focus should be set on pelvic ring stability and less in Matta’s criteria, as THA conversion is a relatively simple procedure to pick up with that complication.

### Conversion to THA

Up to 16% patients with combined injuries may require a conversion to THA [3]. In an analysis of 150 combined injuries, THA conversion in 24 patients was performed 2.2 years after initial trauma [3]. In most cases (88%) due to secondary osteoarthritis, in the remaining cases due to deep infections after hardware removal. THA was more often necessary in patients with involvement of the posterior wall (mainly in transverse PW fractures), hip dislocations, marginal impaction or wall comminution (> 3 pieces) [3]. Interestingly, neither isolated femoral head lesions nor an increased step off in fracture reduction were associated with an increased risk for later conversion to THA. In the majority of the reported cases (55%, an acetabulum first approach was chosen [3].

## Conclusions

The acute treatment of combined acetabular and pelvic ring injuries should follow ATLS algorithms with damage control principles and is similar to pelvic ring injuries without acetabular fractures. The general use of PCCDs, external fixators and C-Clamps cannot be recommended. Aggravating factors may be the presence of hip dislocations and/or femoral shaft fractures (floating hip). Early closed reduction and mechanical fixation of the hip (optionally with external fixators) are mandatory in those cases, the use of binders may even worsen the situation in case of central dislocation of the femoral head, as this may increase the malreduction of the acetabular fracture.

For definitive reduction and fixation of mechanically connected acetabular and pelvic ring injuries (MCAPI) the authors recommend a “pelvic ring first” sequence that starts posteriorly, especially in displaced C-type ring injuries. In case of younger patients, anatomically reducible acetabular fractures and not severely displaced pelvic ring fractures, an “acetabulum first” sequence could be considered. Definitive treatment of pelvic and acetabular fractures should be prioritized and performed within 3–7 days if possible, early total care (ETC) can be considered in selected cases and stable patients.

**Table 1:** Mechanically connected acetabular and pelvic ring injuries (MCAPI). Definition: MCAPI is present if one of these acetabular fracture patterns is associated with one of the pelvic ring pattern on the same side, *Incidence in combined injuries [2, 7, 10]

Acetabulum pattern (Judet)*^#$^	(OTA/AO)	Pelvic ring pattern (OTA/AO)
Transverse (21–61%)	A3.2	B2, B3 (67%)
Both column (14–25%)	A3.3	C1, 2, 3 (23%)
Transverse and posterior wall (7–17%)	B1,2,3	
Anterior column and posterior hemi-transverse (11–13%)	C1,2,3	
T-shape (8–23%)		
Anterior column (4–18%)		

**Fig. 4:**
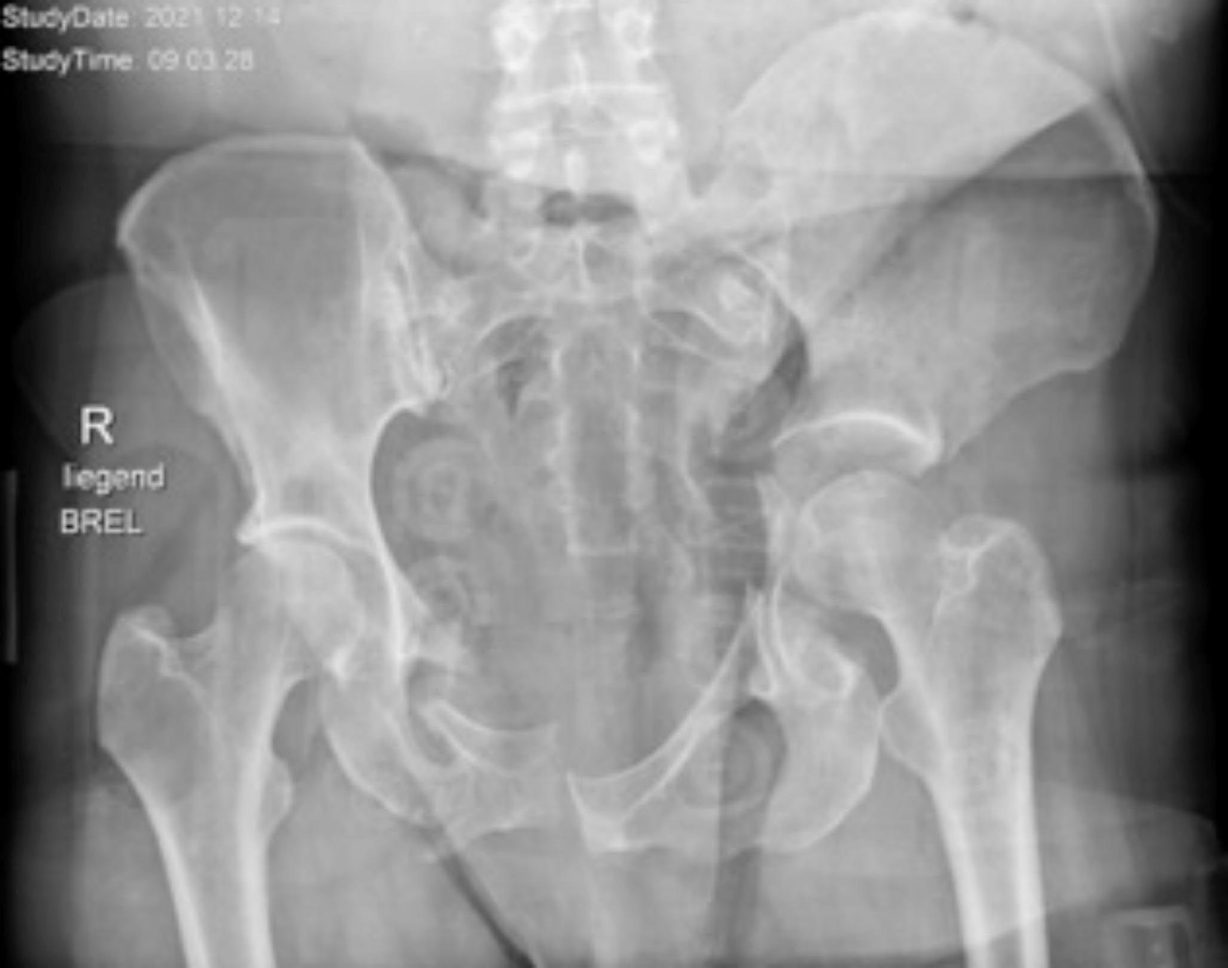
Patient in extremis with 3 C° open pelvic ring fracture, intestinal injury, rupture of external iliac artery and lumbosacral plexus. MCAPI with transverse acetabular pattern and C2 pelvic ring injury on the left side

**Fig. 5:**
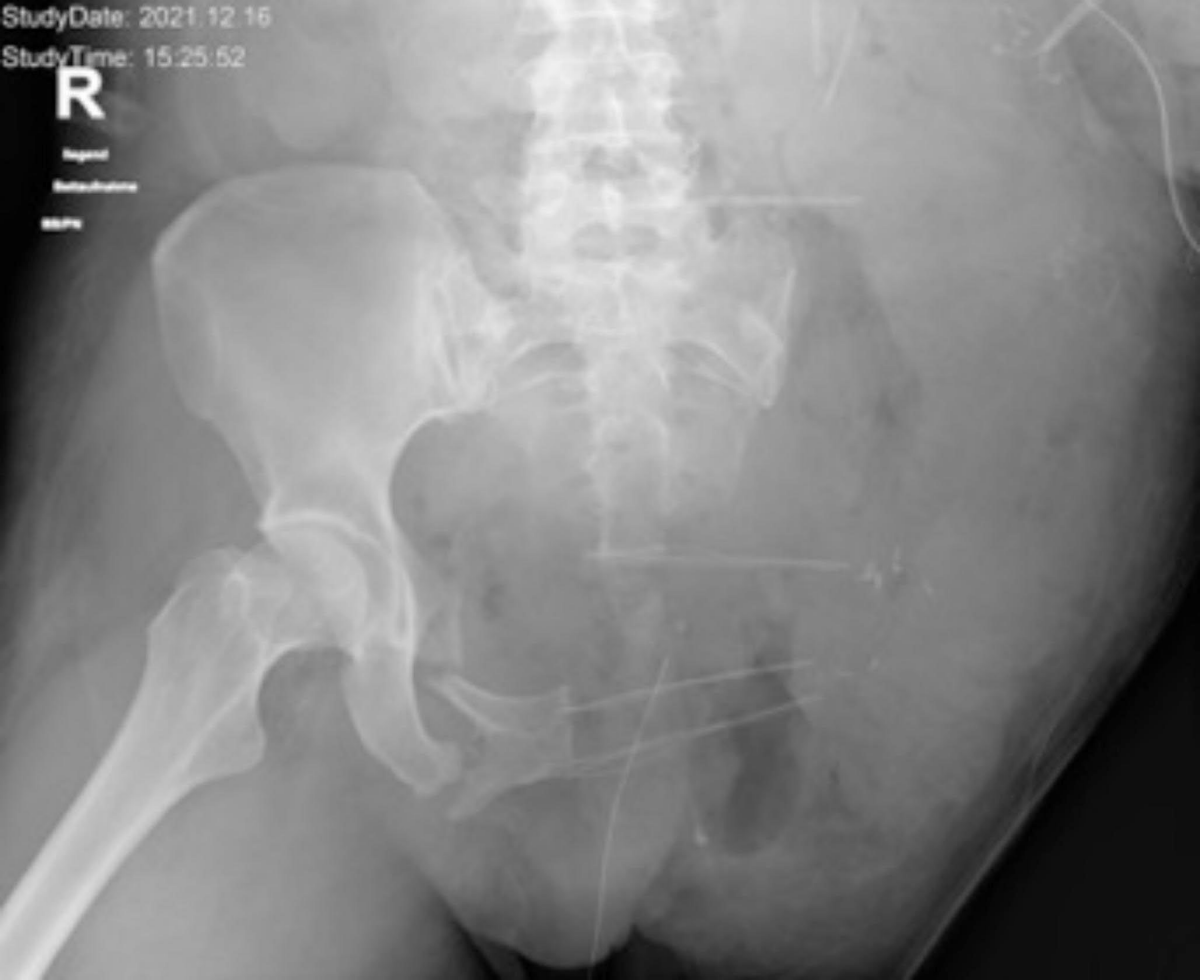
Damage control surgery with salvage procedure. Hemipelvectomy left was performed, patient died 5 days later in sepsis due to massive soft tissue injury and intestinal injuries

**Fig. 6:**
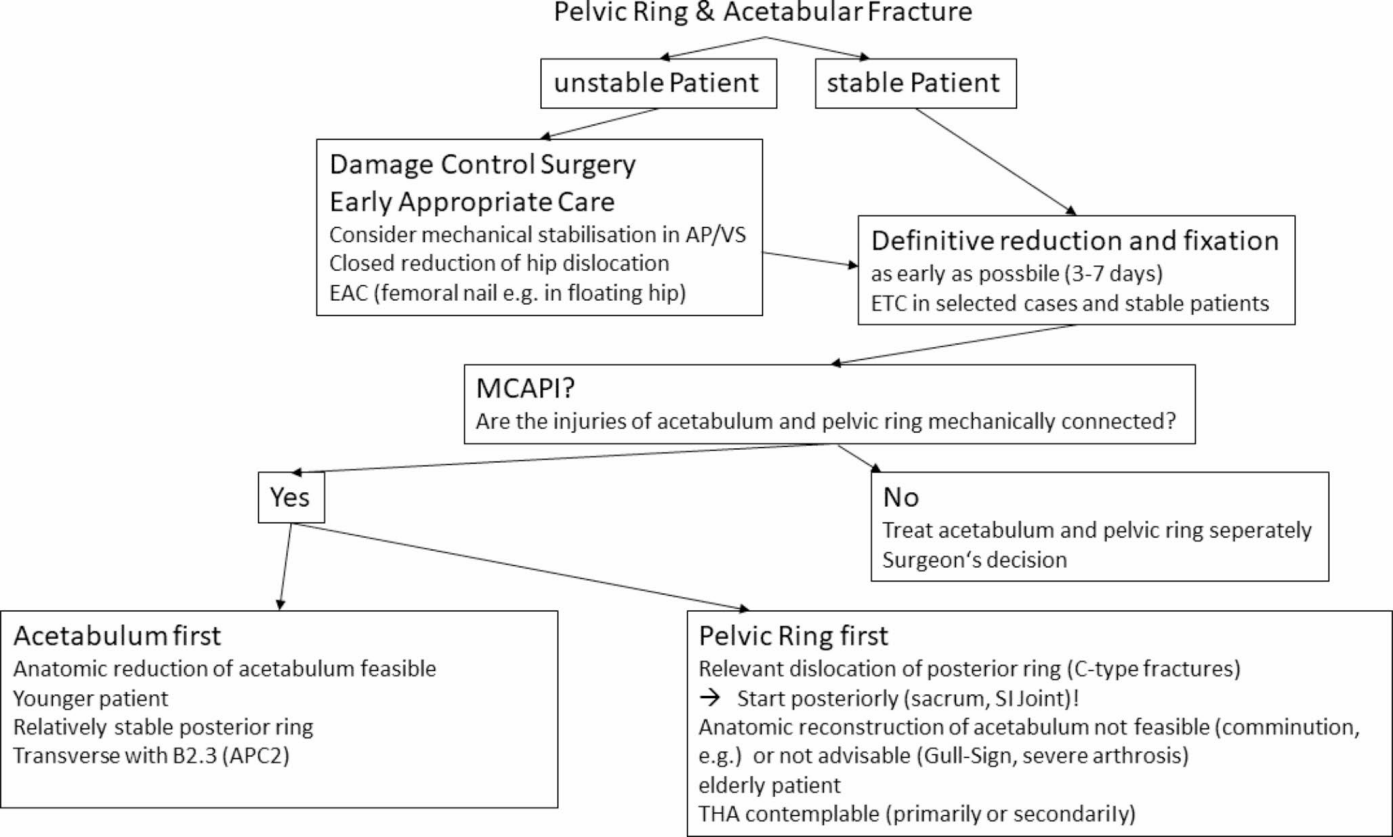
Flowchart for treatment of combined acetabular and pelvic ring injuries (MCAPI and non-MCAPI)
